# Diffusion kurtosis imaging allows the early detection and longitudinal follow-up of amyloid-β-induced pathology

**DOI:** 10.1186/s13195-017-0329-8

**Published:** 2018-01-09

**Authors:** Jelle Praet, Nikolay V. Manyakov, Leacky Muchene, Zhenhua Mai, Vasilis Terzopoulos, Steve de Backer, An Torremans, Pieter-Jan Guns, Tom Van De Casteele, Astrid Bottelbergs, Bianca Van Broeck, Jan Sijbers, Dirk Smeets, Ziv Shkedy, Luc Bijnens, Darrel J. Pemberton, Mark E. Schmidt, Annemie Van der Linden, Marleen Verhoye

**Affiliations:** 1 Bio-Imaging Lab, University of Antwerp, Campus Drie Eiken (CDE-Uc1.14), Universiteitsplein 1, 2610 Antwerp (Wilrijk), Belgium; 2 Janssen Research and Development, Beerse, Belgium; 3 Interuniversity Institute for Biostatistics and Statistical Bioinformatics, Hasselt University, Hasselt, Belgium; 4 Icometrix R&D, Leuven, Belgium; 5 Institute for Biological and Medical Imaging, Technische Universität München, Munich, Germany; 6 DCILabs, Keerbergen, Belgium; 7 HistoGeneX, Antwerpen, Belgium; 8 Expert Group Antwerp Molecular Imaging (EGAMI), University of Antwerp, Antwerp, Belgium; 9 imec-Vision Lab, University of Antwerp, Antwerp, Belgium

**Keywords:** Magnetic resonance imaging, Diffusion tensor imaging, Diffusion kurtosis imaging, Alzheimer’s disease, APP/PS1

## Abstract

**Background:**

Alzheimer’s disease (AD) is a progressive neurodegenerative disorder and the most common cause of dementia in the elderly population. In this study, we used the APP/PS1 transgenic mouse model to explore the feasibility of using diffusion kurtosis imaging (DKI) as a tool for the early detection of microstructural changes in the brain due to amyloid-β (Aβ) plaque deposition.

**Methods:**

We longitudinally acquired DKI data of wild-type (WT) and APP/PS1 mice at 2, 4, 6 and 8 months of age, after which these mice were sacrificed for histological examination. Three additional cohorts of mice were also included at 2, 4 and 6 months of age to allow voxel-based co-registration between diffusion tensor and diffusion kurtosis  metrics and immunohistochemistry.

**Results:**

Changes were observed in diffusion tensor (DT) and diffusion kurtosis (DK) metrics in many of the 23 regions of interest that were analysed. Mean and axial kurtosis were greatly increased owing to Aβ-induced pathological changes in the motor cortex of APP/PS1 mice at 4, 6 and 8 months of age. Additionally, fractional anisotropy (FA) was decreased in APP/PS1 mice at these respective ages. Linear discriminant analysis of the motor cortex data indicated that combining diffusion tensor and diffusion kurtosis metrics permits improved separation of WT from APP/PS1 mice compared with either diffusion tensor or diffusion kurtosis metrics alone. We observed that mean kurtosis and FA are the critical metrics for a correct genotype classification. Furthermore, using a newly developed platform to co-register the in vivo diffusion-weighted magnetic resonance imaging with multiple 3D histological stacks, we found high correlations between DK metrics and anti-Aβ (clone 4G8) antibody, glial fibrillary acidic protein, ionised calcium-binding adapter molecule 1 and myelin basic protein immunohistochemistry. Finally, we observed reduced FA in the septal nuclei of APP/PS1 mice at all ages investigated. The latter was at least partially also observed by voxel-based statistical parametric mapping, which showed significantly reduced FA in the septal nuclei, as well as in the corpus callosum, of 8-month-old APP/PS1 mice compared with WT mice.

**Conclusions:**

Our results indicate that DKI metrics hold tremendous potential for the early detection and longitudinal follow-up of Aβ-induced pathology.

**Electronic supplementary material:**

The online version of this article (DOI: 10.1186/s13195-017-0329-8) contains supplementary material, which is available to authorized users.

## Background

Alzheimer’s disease (AD) is the most common cause of dementia and imposes a serious healthcare burden. Currently, 5.1 million Americans have AD, a number set to double by 2050 [1]. Whilst a tremendous amount of research has been conducted in an attempt to elucidate the pathological factors driving the sporadic form of AD, so far its aetiology remains enigmatic. As a consequence, no real cure yet exists, and currently used drugs are focussed on the management and relief of cognitive symptoms [2]. It is commonly accepted that accurate and early treatment helps to better preserve the patient’s level of function and reduces the societal cost associated with caregiving for patients with AD [3]. Additionally, disease-modifying treatments are expected to delay AD progression optimally when they are administered during the early stages of AD pathology [4]. Therefore, it is of utmost importance to develop the means to detect AD pathology both in an early phase and with high sensitivity.

Currently, a definitive diagnosis of AD can be made only following post-mortem analysis of brain tissue. In contrast, a clinical diagnosis of AD in patients is based on cognitive symptoms, cerebrospinal fluid (CSF) biomarkers and imaging diagnostics [5]. In this context, the assessment of brain atrophy progression by using volumetric magnetic resonance imaging (MRI) has long been considered the most valuable tool for following AD progression [6]. However, although volumetric MRI is indeed a very robust tool to follow this progression, atrophy occurs only late during the disease pathology. As such, volumetric MRI holds little value for translational treatment studies where atrophy is not present. In the present study, however, we focused on amyloid-β (Aβ) plaques, which were previously found to occur much earlier during disease progression, starting decades before actual clinical symptoms became apparent [7]. Aβ originates from amyloid precursor protein and is processed into soluble forms of amyloid-β (sAβ) of various lengths. While normally a physiologically relevant balance exists between sAβ_1–40_ and sAβ_1–42_, in patients with AD, Aβ accumulates in the brain, which eventually results in the formation of oligomeric forms of sAβ. The latter are known to be highly toxic to neuronal synapses and will result in synaptic loss [8]. When left unresolved, these high concentrations of Aβ will also result in deposition of Aβ plaques, which eventually trigger an inflammatory response. Together, all these processes cause extensive remodelling of the brain tissue in regions where Aβ pathology occurs.

Diffusion tensor imaging (DTI) and the more recently developed diffusion kurtosis imaging (DKI) are MRI techniques that are capable of in vivo visualisation of extensive tissue remodelling. Therefore, the usefulness of DTI in the detection of AD pathology is currently being investigated in multi-centre MRI studies. For example, the ADNI2 (Alzheimer’s Disease Neuroimaging Initiative 2) and ADNI-GO (Alzheimer’s Disease Neuroimaging Initiative “Grand Opportunities”) trials included DTI of patients with AD, and they showed that DTI could be a possible biomarker for AD [9]. This inclusion of DTI in human studies is supported by numerous pre-clinical studies conducted in rodents that have shown the ability of DTI to detect amyloidosis. However, the potential of DKI to visualise amyloidosis has been studied somewhat less. DKI provides an estimate of both the Gaussian diffusion distribution (DT metrics) and the deviation of this Gaussian distribution at higher *b* values (DK metrics). The latter makes DKI a more sensitive technique than DTI for visualising microstructural changes [10]. We recently provides proof of principle that DKI is able to detect amyloidosis in mice. By using the APP/PS1 transgenic mouse (a rapidly progressing amyloidosis model [11]), we have previously shown that extensive amyloidosis increases the DK metrics in the cortex and thalamus of 16-month-old APP/PS1 mice as compared with age-matched wild-type (WT) mice [12].

In the present study, we built upon these previous findings and aimed to investigate (1) if DK metrics allow for better separation of APP/PS1 mice from WT than when DT metrics are used, (2) if DKI metrics allow identification of early Aβ-induced pathology and longitudinal follow-up of Aβ plaque-induced pathology, and (3) if the observed changes correlate with the histologically determined pathology.

## Methods

### Animals and experimental design

In this study, male WT C57BL/6 J mice (*n* = 52) and male transgenic APP_KM670/671NL_/PS1_L166P_ mice were used (*n* = 67, referred to as APP/PS1 mice) [11]. Mice were housed in the animal facility of the University of Antwerp during the whole experiment. During the study, mice were kept on a normal 12-h/12-h day-night cycle with ad libitum access to food and water. Additional file 1 shows the weight evolution of the mice in the longitudinal cohort.

**Fig. 1:**
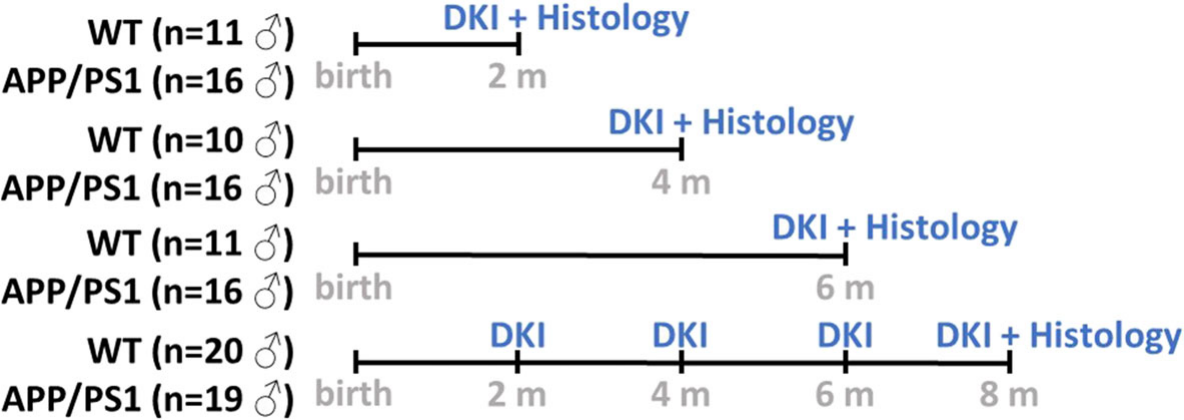
Experimental setup of the study. Diffusion kurtosis imaging (DKI) was performed in three cohorts of male wild-type (WT) and APP/PS1 mice at 2, 4 and 6 months of age, and these mice were sacrificed for histological analysis thereafter. In a fourth, longitudinal cohort of male WT and APP/PS1 mice, we performed DKI at 2, 4, 6 and 8 months of age and thereafter killed these mice for histological analysis

APP/PS1 mice start developing Aβ plaques from the age of 6 to 8 weeks and show aggressive amyloidosis in subsequent months [11]. As such, we acquired the first dataset when mice were 2 months old, when only low Aβ plaque deposition is present (*n* = 20 WT mice and *n* = 19 APP/PS1 mice). We then longitudinally followed these mice by acquiring a DKI datasets when they were 4 and 6 months of age (intermediate Aβ plaque load) and finally at 8 months of age, which corresponds to an extensive Aβ plaque load. After acquisition of this last DKI dataset at 8 months of age, mice were then sacrificed for histological analysis. In addition, three further cohorts of WT and APP/PS1 mice were scanned once each at 2 months (*n* = 11 WT mice and *n* = 16 APP/PS1 mice), 4 months (*n* = 10 WT mice and *n* = 16 APP/PS1 mice) and 6 months (*n* = 11 WT mice and *n* = 16 APP/PS1 mice) of age and killed thereafter for histological analysis. The complete experimental design is shown in Fig. 1.

### MRI scan acquisition

At 2, 4, 6 and 8 months of age, mice were subjected to ^1^H-MRI scanning using a 7-T PharmaScan MRI scanner with a 16-cm diameter horizontal bore (Bruker, Bremen, Germany). This system is equipped with a standard Bruker cross-coil setup using a quadrature volume coil for excitation and an array mouse surface coil for signal detection. The system was interfaced with a Linux PC running TopSpin 2.0 and Paravision 5.1 software (Bruker BioSpin, Ettlingen, Germany). Anaesthesia was induced using 2% isoflurane (Abbott, Maidenhead, UK) in a gas mixture of 30% O_2_ and 70% N_2_ at a flow rate of 600 ml/minute. During MR image acquisition, the isoflurane concentration was initially set at 2% and subsequently lowered when required to maintain a stable respiration rate of (110 ± 10) breaths per minute, which was monitored using a pressure-sensitive pad. In addition, body temperature was monitored via a rectal probe and was held constant between 37.0 °C and 37.3 °C using warm air coupled to a feedback unit (SA Instruments, Stony Brook, NY, USA). PC-sam monitoring software (SA Instruments) was used to measure respiration rate and to measure and control body temperature. Following MR image acquisition, mice were left to recover separately under a heating lamp before being returned to their respective cages.

To ensure uniform slice positioning, we first acquired axial, sagittal and horizontal multi-slice 2D rapid acquisition and relaxation enhancement (RARE) images using the following parameters: repetition time (TR) = 2500 ms, echo time (TE) = 33 ms, matrix size (256 × 256), field of view (FOV) = (20 × 20) mm^2^, resolution = (0.078 × 0.078) mm^2^, nine slices, slice thickness = 0.8 mm, and RARE factor = 8. Following correct positioning, we acquired three DKI scans, each with 20 unique diffusion gradient directions and seven *b* values (400, 800, 1200, 1600, 2000, 2400 and 2800 s/mm^2^). In addition, seven images without diffusion weighting (*b*_0_) were included for each DKI scan. This yielded DKI data comprising 21 *b*_0_ images and diffusion weighting for 60 diffusion directions with seven *b* values. Images were collected with a multi-slice two-shot spin-echo/echo planar imaging sequence with the following parameters: TR = 7000 ms, TE = 23.25 ms, δ = 4 ms, Δ = 12 ms, acquisition matrix = (96 × 96), spatial resolution = (0.214 × 0.214) μm^2^, 28 horizontal slices, and slice thickness = 0.20 mm. This resulted in a total acquisition time of 1 h, 43 minutes. Next, a high-resolution 3D anatomical image was acquired using a T2-weighted 3D RARE sequence in the same horizontal orientation as the DKI data. The following parameters were used: TR = 3185 ms, TE = 44 ms, and spectral width = 50 kHz, averages = 1, RARE factor = 8, matrix size = (265 × 64 × 50), FOV (20.5 × 13.0 × 10.0) mm^3^, resolution = (0.080 × 0.203 × 0.200) mm^3^, and total acquisition time = 21 minutes.

### MRI analysis

Prior to the actual image analysis, we performed a visual inspection for quality of the acquired raw data (e.g., ghosting and/or movement) combined with a semi-automated quality control to avoid bias in the diffusion parameter estimation due to MR acquisition artefacts. This semi-automated data quality control consisted of (1) validating the signal decay with increasing *b* values, (2) validating if signals obtained with high *b* values did not reach the noise level, (3) validating the magnitude of the signal-to-noise ratio, and (4) validating the parametric estimation error using a chi-square test (*see below*). Once quality was assured, image pre-processing and analysis were initiated. The data were corrected for motion and eddy current artefacts using the Functional Magnetic Resonance Imaging of the Brain (FMRIB) Software Library (FSL) [13].

The DKI model is shown below:

1 $$ {S}_{dki}\left(b,g\right)=S(0)\exp \left(-b{\sum}_{i,j=1}^3{g}_i{g}_j{D}_{ij}+\frac{b^2}{6}{\left({\sum}_{i=1}^3\frac{D_{ii}}{3}\right)}^2{\sum}_{i,j,k,l=1}^3{g}_i{g}_j{g}_k{g}_l{W}_{ij kl}\right) $$

with *S*(0) being the signal intensity without diffusion weighting, *D* being the rank 2 diffusion tensor, *W* being the rank 4 kurtosis tensor, *b* being the *b* value, and *g* being the diffusion gradient direction. The DKI model was voxel-wise fitted to the diffusion-weighted images. The diffusion tensor and kurtosis tensor quantify the Gaussian diffusion profile and the deviation from a Gaussian diffusion distribution, respectively [10, 14]. The diffusion tensor and the diffusion kurtosis tensor were estimated simultaneously using conditional least squares estimators while imposing positivity on the kurtosis coefficients [15]. The conditional least squares estimator explicitly accounts for the Rician MR data distribution, for which the noise level has been estimated [16]. Rotational invariant parameter maps of axial diffusivity, radial diffusivity (RD), mean diffusivity (MD) and fractional anisotropy (FA) were computed from the diffusion tensor, and further refered to as diffusion tensor (DT) metrics, and axial kurtosis (AK), radial kurtosis (RK) and mean kurtosis (MK) maps were computed from the diffusion kurtosis tensor, and further refered to as diffusion kurtosis (DK) metrics, using MATLAB software (Mathworks Inc., Natick, MA, USA) [14, 17].

A study-based atlas was constructed with Advanced Normalization Tools [18] software using 25 randomly selected 3D T2-weighted MRI datasets across both genotypes and all ages. We delineated 23 grey matter and white matter regions of interest (ROIs) on this atlas using AMIRA (version 5.4) (Additional file 2). All individual 3D T2-weighted MRI scans were then normalised to the atlas. The inverse transformation of the normalisation was used to map the atlas ROIs onto the native space of the individual 3D T2-weighted MRI scans. Afterwards, all individual *b*_0_ MR images were co-registered to their corresponding 3D T2-weighted MRI scans using FSL software. Using the inverse transformation of the latter, we mapped the individual 3D ROIs to the individual DT and DK metric maps. On the basis of optimal contrast of the FA map to differentiate grey matter and white matter, we finally manually checked and, if necessary, corrected the contours of the grey matter and white matter ROIs to limit a possible partial volume effect. Finally, ROI-averaged DT and DK metrics (MD, axial diffusivity, RD, FA and MK, AK and RK) were extracted from the respective metric maps.

### Histological analysis

For histological examination, mice were sacrificed by means of cervical dislocation. The complete brain was dissected and fixed in Fade4 fixative. Fixed brains were sent to HistoGeneX (Antwerp, Belgium), where 5-μm-thick sagittal paraffin-embedded sections were cut from the left hemisphere. Sectioning was started at the middle of the brain, and ten sagittal sections were acquired at 150-μm intervals, covering the whole hemisphere. Immunohistochemical (IHC) staining for Aβ plaques was initiated by depigmenting slides using potassium permanganate for 3 minutes, followed by oxalic acid for 1 minute. Slides were pre-treated in formic acid for 10 minutes to retrieve epitopes and were then incubated for 15 minutes at room temperature with a mouse anti-Aβ (clone 4G8) antibody (1:20,000, SIG-39200; Eurogentec, Angers, France). Next, slides were incubated with a labelled polymer (Dako EnVision + System-HRP Labeled Polymer Anti-Mouse, K4001; Dako). Finally, the substrate was visualised using 3,3′-diaminobenzidine (DAB) chromogen (Dako Liquid DAB+ substrate chromogen system; Dako) for 5 minutes. All steps were performed using the automated Lab Vision Autostainer 480S (Thermo Scientific, Waltham, MA, USA). In every 4G8 staining run, an immunoglobulin G (IgG) control (mouse IgG2b; Dako) was also included. For myelin basic protein (MBP) staining, epitope retrieval was performed in Target Retrieval Solution (Dako) for 30 minutes at 97 °C. After endogenous peroxidase activity was quenched, the slides were incubated for 30 minutes at room temperature with a mouse anti-MBP (clone SMI-94) antibody (1:5000, SMI-94R; Covance Antibody Products, Princeton, NJ, USA). Next, slides were incubated with a labelled polymer (Dako EnVision + System-HRP Labeled Polymer Anti-Mouse, K4001). Finally, the substrate was visualised using DAB chromogen for 5 minutes. All steps were performed on the automated Lab Vision Autostainer 480S. In every MBP staining run, an IgG control (mouse IgG1; Abcam, Cambridge, UK) was included. For microgliosis (ionised calcium-binding adapter molecule 1 [IBA1]) IHC staining, epitope retrieval was performed in citrate buffer (pH 6; Lab Vision) for 30 minutes at 97 °C. After quenching endogenous peroxidase activity, the slides were incubated for 30 minutes at room temperature with a rabbit anti-IBA1 antibody (1:5000, 019-19741; Wako Pure Chemical Industries, Osaka, Japan). Next, slides were incubated with a labelled polymer (Dako EnVision + System-HRP Labelled Polymer Anti-Rabbit, K4003). Finally, the substrate was visualised using the DAB chromogen (Dako Liquid DAB+ substrate chromogen system) for 5 minutes. All steps were performed using the automated Lab Vision Autostainer 480S. In every IBA1 staining run, an IgG control (rabbit IgG; Dako) was included. For astrogliosis (glial fibrillary acidic protein [GFAP]) IHC staining, epitope retrieval was performed using cell conditioning solution (Ventana Medical Systems, Tucson, AZ, USA). The slides were incubated for 28 minutes at 37 °C with a rabbit anti-GFAP antibody (1:7500, Z0334; Dako). Next, slides were incubated with an OmniMap anti-rabbit HRP detection system (Ventana Medical Systems). All steps were performed on the automated Ventana Discovery® XT platform (Ventana Medical Systems). In every GFAP staining run, an IgG control (rabbit IgG; Dako) was also used. For all four staining runs, stained slides were scanned using the MIRAX digital slide scanner (Carl Zeiss Microscopy, Göttingen, Germany).

### Quantification of histology

Quantification of the stained slides was performed by DCILabs (Keerbergen, Belgium). In short, the histological images are colour de-convolved to separate the colours on the stained slides [19]. This generates a grey value image of the MBP-, GFAP-, 4G8- and IBA1-stained slides. Hysteresis thresholds were applied on these images to determine the percentage of area positive for the respective staining (percent optical density [%O.D.]) and for each of these ROIs. To quantify the amount of 4G8-positive Aβ plaques, a mask based on an intensity cutoff value to distinguish Aβ plaques from background signal in the grey value images was compiled, which then automatically counted the amount of objects in the mask. To assess the presence of elongated structures in the image, an anisotropic measure was defined as the ratio of the difference and the sum of the eigenvectors of the local structure tensor [20]. All analyses were performed with software developed in-house using OpenSlide C interface (openslide.org) to read the MIRAX images.

### 3D stacking of histological images and co-registration to MRI

**Fig. 2:**
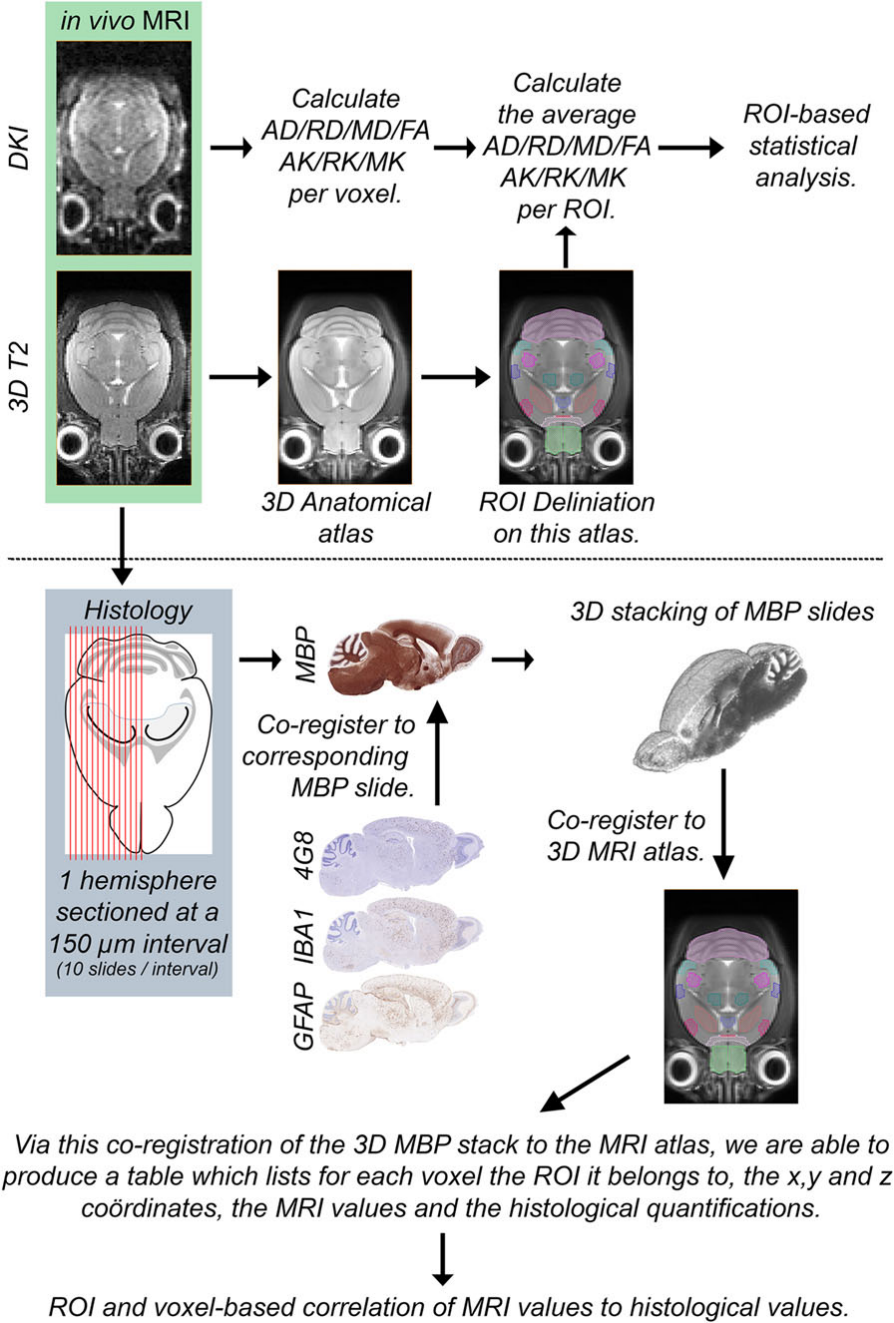
Schematic overview of the data acquisition and analysis pipeline used in this study. *AD* Axial diffusivity, *AK* Axial kurtosis, *DKI* Diffusion kurtosis imaging, *FA* Fractional anisotropy, *4G8* Anti-amyloid-β (clone 4G8) antibody, *GFAP* Glial fibrillary acidic protein, *IBA1* Ionised calcium-binding adapter molecule 1, *MBP* Myelin basic protein, *MD* Mean diffusivity, *MK* Mean kurtosis, *MRI* Magnetic resonance imaging, *RD* Radial diffusivity, *RK* Radial kurtosis, *ROI* Region of interest

Co-registration of all images of the histological staining was performed, and an overview of the whole co-registration pipeline is shown in Fig. 2. In short, because the MBP staining contains considerable anatomical information, we used the MBP-stained slide of each sectioned interval (150 μm apart) to create a 3D histological reference space. These MBP-stained slides were stacked onto a 3D dataset using an algorithm previously described by Lowe et al. [21] and Ourselin et al. [22]. Next, we co-registered this 3D histological stack to the 3D T2-weighted MRI atlas as described by Ourselin et al. [22] and Modat et al. [23]. Next, a moment-matching algorithm was used to match the 4G8, GFAP and IBA1 staining with the MBP images in order to propagate all histological stains to the corresponding MR images. We then combined all MRI and histological data and created a relational database which contained the following per voxel: the MRI atlas coordinates, the ROI information, all diffusion and diffusion kurtosis metrics, and the corresponding histological values. On the basis of this database, we performed the statistical analyses described below.

### Statistical analysis

#### ROI-based MRI analysis

To evaluate significant differences for each ROI between the different genotypes (WT and APP/PS1 mice), as well as over the different ages of the mice that were followed longitudinally, we used a marginal model with age- and genotype-specific fixed effects. The latter models the evolution of all DT and DK metrics over time while taking into account the association between these DTI and DKI metrics of any given subject at any given age [24–26]. A Bonferroni correction was applied for the different ages.

#### Linear discriminant analysis

Given a set of MRI parameters (MK, AK, RK, MD, axial diffusivity, RD and FA), the linear discriminant analysis (LDA) algorithm searches for a linear combination of MRI parameters for which the genotype misclassification error (MCE; the proportion of incorrectly classified mice) is minimised. The LDA was done using only the DT metrics (MD, axial diffusivity, RD and FA), only the DK metrics (MK, AK and RK) or a combination of both DT and DK metrics. Two-fold cross-validation was performed by randomly splitting the data into a training group and a test group. The training group was composed by randomly selecting ten WT mice and ten APP/PS1 mice. The test group contained the remaining ten WT mice and nine APP/PS1 mice. This process was repeated 1000 times, and the test data MCE was computed for each iteration. The MCE denotes the proportion of mice in the test group assigned to the wrong genotype [27]. To test whether the MCEs were significantly different, we performed a pairwise Kolmogorov-Smirnov test, and the Bonferroni correction was applied for the three tests performed (DT vs DK, DK vs DT + DK and DT vs DT+ DK).

#### Least absolute shrinkage and selection operator analysis

To identify which MRI metric contributed the most to the correct classification of the genotype, we applied least absolute shrinkage and selection operator (LASSO) logistic regression [28]. In short, we randomly divided all mice into ten distinct groups. A classifying model is trained using MRI metrics from nine of these groups, and the resulting model is applied to the remaining group to determine how good the classifying model is at determining the correct genotypes with different weights of L_1_ regularisation. We repeated this ten times, withholding a different dataset each time, to achieve a ten-fold cross validation. The result of LASSO analysis indicates if an MRI metric was retained as a classifier (1) or not (0). Moreover, because the 10 distinct groups were chosen randomly, we repeated this whole procedure 100 times. For each metric, we then calculated the percentage of the times it was used to classify the genotypes as an indication of the importance of each metric to correctly classify the genotype.

#### ROI-based correlation between MRI and histology

To evaluate the potential of the DT and DK metrics as possible markers for the histological features of the motor cortex, we calculated the respective Pearson’s correlation coefficients. A Bonferroni multiplicity correction was applied for the different parameters investigated, and a multiplicity-corrected *p* value < 0.05 was considered significant.

#### Voxel-based statistical parametric mapping

To assess voxel-level differences between WT and APP/PS1 mice, we co-registered every individual 3D T2-weighted MR image to the 3D T2-weighted MRI atlas which we had previously constructed. We then transformed all DT and DK metric maps to this 3D T2-weighted MRI atlas and spatially smoothed these maps using a Gaussian kernel. Next, statistical analysis was done for each DT and DK metric separately, using statistical parametric mapping [29]. A false discovery rate correction for multiple comparisons was performed [30], and only voxels with *p* < 0.05 were visualised.

#### Voxel-based correlation between MRI and histology

We applied a Bayesian multivariate linear regression [31] to estimate the predictive value of the DT metrics, the DK metrics, or a combination of the DT and DK metrics for the histological outcome at a voxel-based level. Evaluation was done using a leave-one-animal-out methodology. Prediction of the histological values based on the DT or DK metrics of a test mouse was done using a model that was trained on the basis of data of all mice except the test mice. The latter was then validated by cross-validating the correlation coefficients, and the average of the correlation coefficients was calculated after applying a Fisher Z-transformation [32].

## Results

### ROI-based analysis of diffusion and DKI metrics

**Fig. 3:**
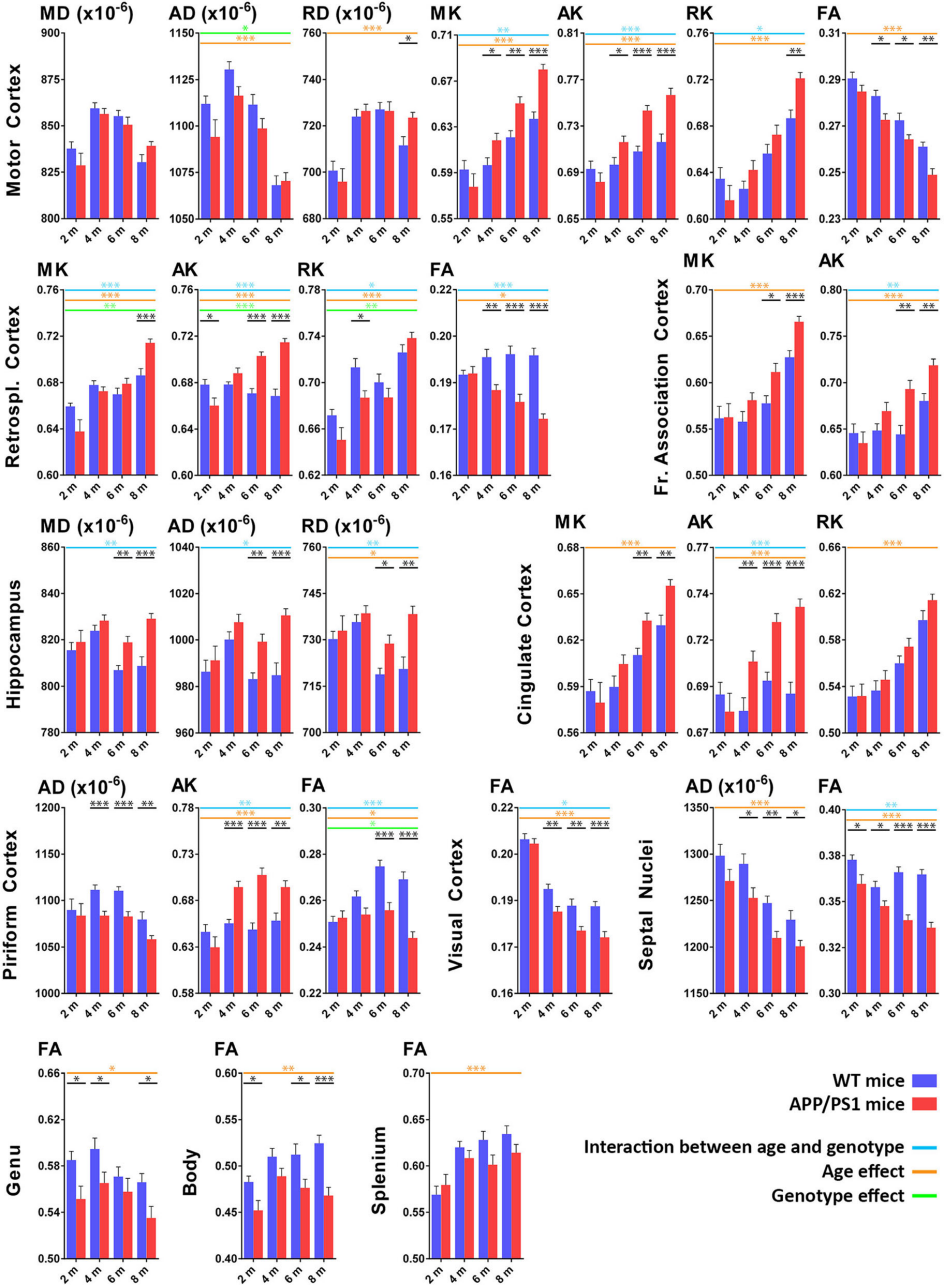
Region of interest (ROI)-based DKI analysis. The most interesting differences in diffusion tensor and diffusion kurtosis metrics of several grey and white matter ROIs. We show only data from the longitudinal cohort of wild-type WT (blue bars) and APP/PS1 mice (red bars) at 2, 4, 6 and 8 months of age. Shown are the mean diffusivity (MD), axial diffusivity (AD) and radial diffusivity (RD); the mean kurtosis (MK), axial kurtosis (AK) and radial kurtosis (RK); and the fractional anisotropy (FA). Significant differences between WT and APP/PS1 mice at any given time point are shown in *black*; genotype effect is indicated by *green lines*; age effect is indicated by orange lines; and interaction between age and genotype is indicated by *blue lines*. *p < 0.05, **p < 0.01 and ***p < 0.001

We have previously shown that DKI is able to visualise Aβ plaque-induced pathology in APP/PS1 mice at 16 months of age [12]. In the present study, we first investigated the ability of DKI to visualise early and progressive Aβ plaque-induced pathology in APP/PS1 mice at 2, 4, 6 and 8 months of age. As such, we performed an ROI-based analysis of the longitudinally acquired dataset. The most meaningful statistically significant differences for different metrics of different ROIs are shown in Fig. 3.

In the motor cortex, we observed a genotype effect for axial diffusivity and an age effect for axial diffusivity and RD. In addition, we observed an increased RD in APP/PS1 mice as compared with WT mice at 8 months of age. Compared with these DT metrics, however, greater significant differences were observed for the DK metrics. As such, compared with WT mice, APP/PS1 mice showed (1) higher MK values at 4, 6 and 8 months of age; (2) higher AK values at 4, 6 and 8 months of age; (3) higher RK values at 8 months of age; and (4) lower FA values at 4, 6 and 8 months of age. Additionally, we observed an age effect and an interaction between age and genotype for MK, AK and RK. For the retrosplenial cortex, the MK, AK and RK values showed an age effect, a genotype effect, and an interaction between age and genotype. However, the increased AK and decreased FA values of APP/PS1 mice compared with WT mice were the only straightforward differences which persisted over time and became larger as pathology progressed. Albeit to a lesser degree, this trend of increased MK and AK values in APP/PS1 mice compared with WT mice was also observed in the frontal association cortex. The AK values of the cingulate cortex did show an age effect, as well as an interaction between age and genotype, and were significantly increased at 4, 6 and 8 months of age in APP/PS1 mice compared with WT mice. In contrast, in the hippocampus, we did not observe any significant changes in DK metrics, but we did observe significant changes in the DT metrics. MD, axial diffusivity and RD were significantly increased in APP/PS1 mice as compared with WT mice at 6 and 8 months of age, and an interaction between genotype and age was observed for all three DT metrics. Next, from 4 months of age onwards, the piriform cortex showed significantly reduced axial diffusivity but increased AK in APP/PS1 mice compared with WT mice. In addition, an age effect and an interaction between age and genotype were noted for AK. For both the piriform cortex and the visual cortex, a significantly decreased FA value in the APP/PS1 mice was noted as compared with WT mice, respectively from month 6 and month 4 onwards. Additionally, for the FA values of the piriform cortex, we observed an age effect, a genotype effect and an interaction between age and genotype. In the septal nuclei, we observed decreased axial diffusivity in APP/PS1 mice as compared with WT mice, which was significant from month 4 onwards. More importantly, however, an age effect and an interaction between age and phenotype were observed in addition to a progressive decrease of the FA values in APP/PS1 mice as compared with WT mice, which were significant at all ages. Lastly, we observed a decrease in FA values in APP/PS1 mice compared with WT mice in all three parts of the corpus callosum: the genu, the body and the splenium. While this was significant at 2, 4 and 8 months of age in the genu, as well as at 2, 6 and 8 months of age in the body, the splenium showed a trend only towards decreased FA values in APP/PS1 mice.

### LDA and LASSO analysis of the motor cortex

**Fig. 4:**
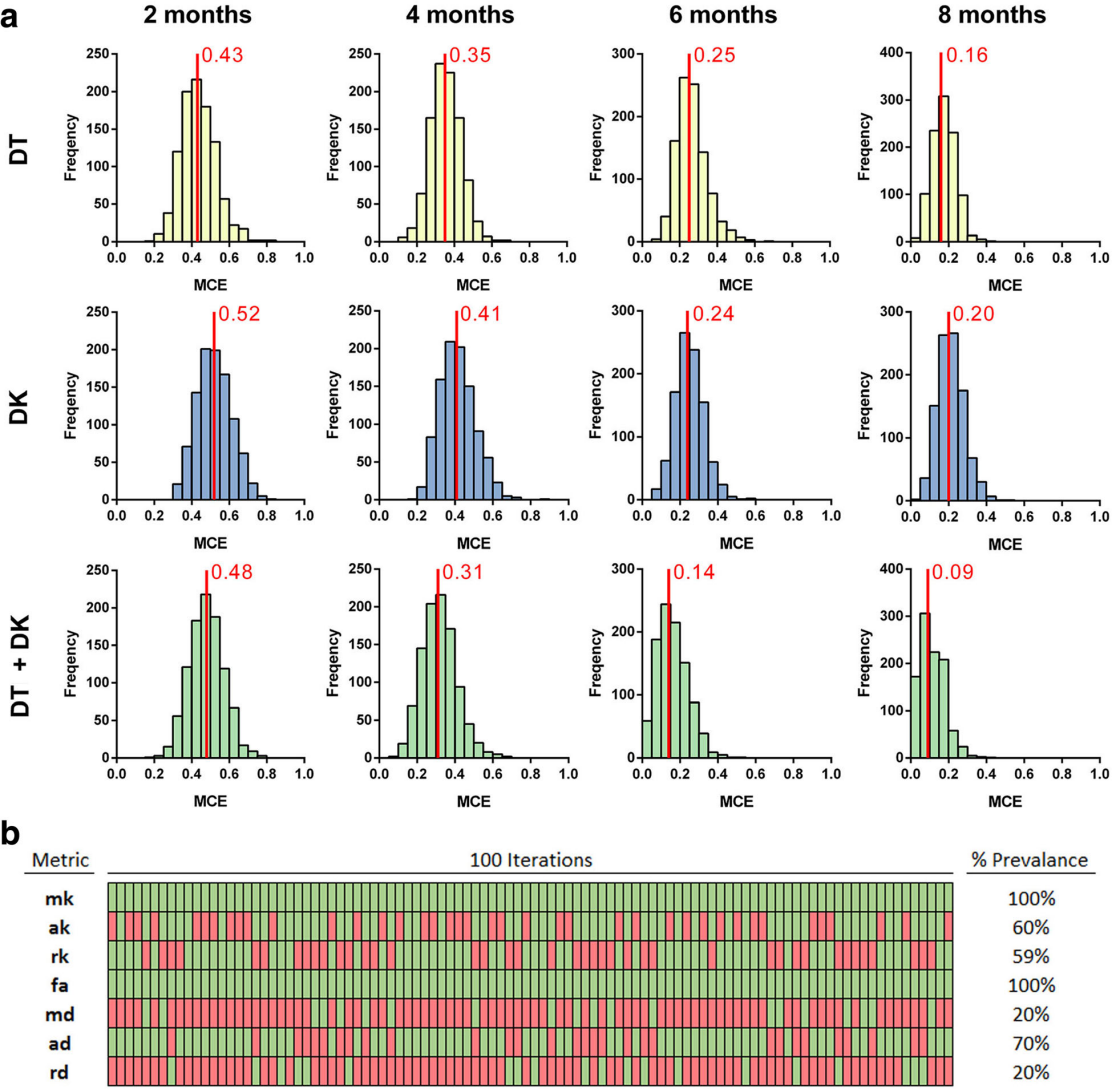
Linear discriminant analysis and least absolute shrinkage and selection operator (LASSO) analysis of the motor cortex. **a** The frequency distribution histograms of the misclassification error (MCE) of the motor cortex are shown (percentage of animals which were attributed the wrong genotype). The MCE is calculated at 2, 4, 6 and 8 months of age when using only the diffusion tensor (DT) metrics (*top row*), the diffusion kurtosis (DK) metrics (*middle row*) or a combination of the DT and DK metrics (*bottom row*). The *red lines* indicate the average MCE. **b** LASSO analysis was done to determine which of the DTI or DKI metrics contribute the most to a correct classification of the genotype. The heat map shows all 100 iterations of the LASSO analysis for all metrics, where *green* indicates that the metric was used and *red* indicates that the metric was not used. On the *right*, we show the percentage of times that the metric contributed to a correct genotype classification in all of these 100 iterations (percent prevalence)

Because the ROI-based analysis showed differences between APP/PS1 mice and WT mice in multiple ROIs, we investigated if we could successfully predict the genotype of the mice on the basis of statistical linear discriminant modelling of the present data. For this purpose, we limited our analysis to the motor cortex because this region showed the most statistically significant differences in the ROI-based analysis (*see above*), and the DK metrics of the motor cortex correlated well with the histologically determined pathology (*see below*). In Fig. 4a, we show the MCE (percentage of animals assigned to the wrong genotype) of the LDA at 2, 4, 6 and 8 months of age when using only the DT metrics (*top row*), the DK metrics (*middle row*) or the DT and DK metrics combined (*bottom row*). At 2 months of age, the MCE for the DT metrics (0.43), the DK metrics (0.52) or the DT and DK metrics combined (0.48) indicates the inability of all three metric combinations to separate APP/PS1 mice from WT mice. However, the MCE decreases with age for all three metric combinations, and as such, at 8 months of age, the MCE for the DT metrics is 0.16, and for the DK metrics, it is 0.20. However, when we combined DT and DK metrics, the MCE was 0.09. When comparing the MCE of these three metric combinations at 8 months of age, we could show that these are significantly different from each other with a *p* value < 0.0001 for any of the three possible comparisons (DT vs DK, DT vs DT + DK, and DK vs DT+ DK). Taken together, this clearly indicates the added value of acquiring DKI data and using all metrics estimated.

Because the combination of both DT and DK metrics gave the lowest MCE, we performed LASSO analysis to investigate which of these metrics were the most important for a correct classification of the genotype. In Fig. 4b, we show a heat map of all 100 iterations of the LASSO analysis, which were done on the data of the 8-month-old mice. On one hand, the MK and FA are the most important metrics for a correct classification of the genotype because they were involved in 100% of the iterations. On the other hand, AK, RK and axial diffusivity were involved in 60%, 59% and 70% of the iterations, respectively. In contrast, MD and RD were involved in just 20% of the iterations. These results clearly indicate that, despite all seven metrics contributing to a different degree, a correct genotype classification depended foremost on the DK metrics and FA.

### Correlation between ROI-based DKI parameters and histology

**Fig. 5:**
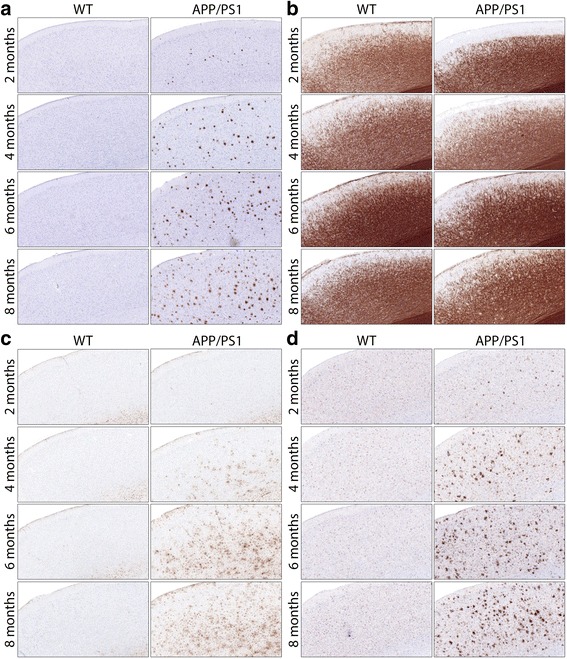
Representative histological images of the motor cortex of the wild-type (WT) and APP/PS1 mice (*left* and *right columns*, respectively) at 2, 4, 6 and 8 months of age (*top* to *bottom rows*). The different panels show the anti-amyloid-β (clone 4G8) antibody staining for amyloid-β plaques (**a**), the myelin basic protein staining for myelin basic protein (**b**), the glial fibrillary acidic protein staining for astrogliosis (**c**) and the ionised calcium-binding adapter molecule 1staining for microgliosis (**d**)

Following the ROI-based analysis, we wondered if the changes observed in DKI metrics correlated with the Aβ-driven pathological changes in the tissue microstructure. Therefore, we sliced the full left hemisphere and collected brain tissue slides at a 150-μm intervals, allowing us to create 3D histological stacks of the performed staining. In Fig. 5, we show a representative close-up of the motor cortex for both WT and APP/PS1 mice at each of the four ages. While these images are only qualitative, it can already be appreciated that the Aβ-induced pathology progresses as APP/PS1 mice become older, as shown by an increased intensity of 4G8, GFAP and IBA1 staining (Fig. 5a, c and d, respectively) and a decreased intensity of the MBP staining (Fig. 5b).

**Fig. 6:**
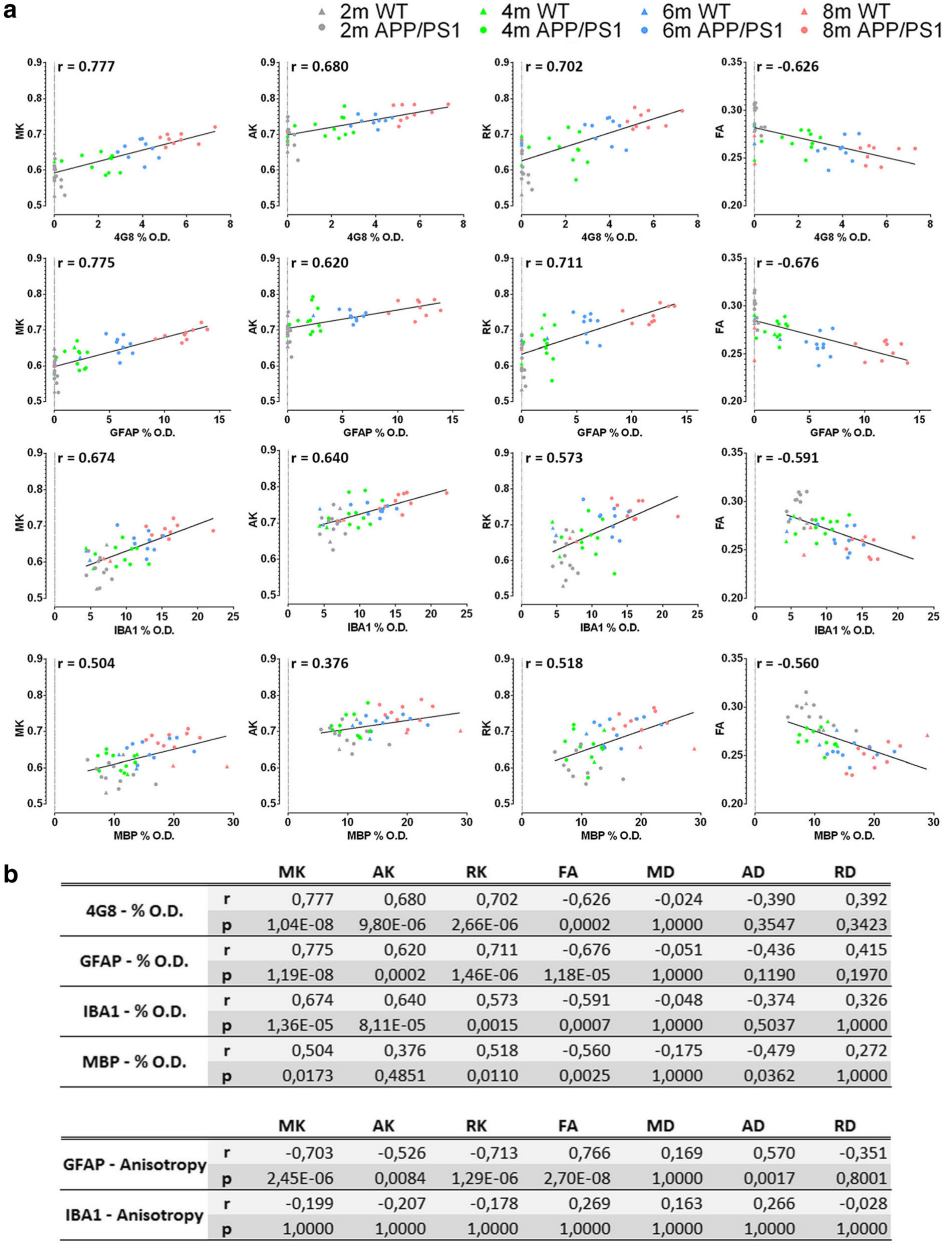
Region of interest-based histological correlation analysis. **a** Graphs showing the Pearson correlations between mean kurtosis (MK), axial kurtosis (AK), radial kurtosis (RK) and fractional anisotropy (FA) and percent optical density (%O.D.) of anti-amyloid-β (clone 4G8) antibody (4G8), glial fibrillary acidic protein (GFAP), ionised calcium-binding adapter molecule 1 (IBA1) and myelin basic protein (MBP). The graph includes data from the wild-type (WT) mice (*triangles*) and APP/PS1 mice (*circles*) at 2 months of age (*grey*), 4 months of age (*green*), 6 months of age (*blue*) and 8 months of age (*red*). **b** Pearson correlation values (*r*) and *p* values of these correlations between the different diffusion tensor and diffusion kurtosis metrics and the histological parameters

Next, we statistically modelled the ability of all DT and DK metrics to predict the corresponding histological parameters in the motor cortex. In Fig. 6a, we show the correlation graphs of the MK, AK, RK and FA metrics with the %O.D. 4G8, GFAP, IBA1 and MBP. The graphs of the correlations of MD, axial diffusivity and RD with these histological parameters are provided in Additional file 3, and the *r* and *p* values of all these correlations are shown in Fig. 6b. MK, AK and RK are positively correlated with the %O.D. 4G8, GFAP and IBA1 in a highly significant manner. MK and RK also correlate with the %O.D. MBP, albeit to a lesser degree. FA shows a significant negative correlation with the %O.D 4G8, GFAP, IBA1 and MBP. In contrast, the MD, axial diffusivity and RD metrics do not correlate with the %O.D. 4G8, GFAP, IBA1 and MBP (with the exception of axial diffusivity, which correlates negatively with the %O.D. MBP). This indicates that DK metrics correlated with the underlying Aβ-induced pathology, whereas DTmetrics did not correlate with Aβ-induced pathology. In Fig. 6b, we also show the *r* and *p* values of the correlations between MK, AK, RK, FA, MD, axial diffusivity and RD and the GFAP and IBA1 anisotropy values. The latter is a readout for the ramification of GFAP-positive astrocytes and IBA1-positive microglia. MK, AK and RK were significantly negatively correlated with GFAP anisotropy, whereas FA and axial diffusivity were significantly positively correlated with GFAP anisotropy. In contrast, MD and RD did not correlate with GFAP and IBA1 anisotropy. We did not observe any correlations between DT or DK metrics and IBA1 anisotropy.

### Voxel-based statistical parametric mapping of DKI parameters

**Fig. 7:**
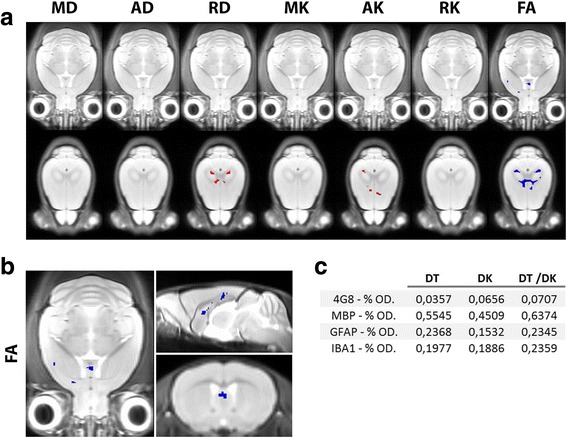
Voxel-based statistical parametric mapping. **a** Statistical parametric maps at two different levels in the brains of mice at 8 months of age. On these images, for each of the seven metrics (mean diffusivity [MD], axial diffusivity [AD], radial diffusivity [RD], mean kurtosis [MK], axial kurtosis [AK], radial kurtosis [RK] and fractional anisotropy [FA]), the voxels with a decreased value in APP/PS1 mice compared with wild-type (WT) mice are shown in *blue*, and the voxels with an increased value in APP/PS1 mice compared with WT mice are shown in *red*. Only voxels with a false discovery rate-corrected *p* > 0.05 are shown. **b** Close-up of the FA statistical parametric map showing reduced FA in APP/PS1 mice in the septal nuclei at 8 months of age (*blue voxels*). **c** Voxel-based correlation values between the percent optical density (%O.D.) anti-amyloid-β (clone 4G8) antibody (4G8), myelin basic protein (MBP), glial fibrillary acidic protein (GFAP) and ionised calcium-binding adapter molecule 1 (IBA1) and the diffusion tensor metrics (DT), the diffusion kurtosis metrics (DK), and the DT and DK metrics (DT/DK)

To compare the ROI-based analyses with a voxel-based approach (which is often used in human studies), we performed statistical parametric mapping, and the resulting images of the mice at 8 months of age are shown in Fig. 7a. We did not observe differences at 2 and 4 months of age, and we observed only very limited differences at 6 months of age (data not shown). Red voxels indicate an increased value in the APP/PS1 mice as compared with the WT mice, whilst blue voxels indicate a decreased value in the APP/PS1 mice as compared with the WT mice. At 8 months of age, mice showed reduced FA in the septal nuclei (close-up shown in Fig. 7b). We also observed reduced FA and an increase in RD in the corpus callosum.

### Voxel-based correlation between DKI parameters and histology

Next, we determined how well DKI metrics predict the underlying pathology at a voxel-based level. As described earlier, we created 3D histological stacks of the different histological stains, which were then co-registered to the corresponding 3D T2-weighted MRI image and, via this intermediate step, thus also to the DKI images. In Fig. 7c, we show the motor cortex average multivariate cross-validated correlation values (of all 8-month-old WT and APP/PS1 mice combined) between the percentage of area stained of the four different histological stains and the DKI metrics (combining all DT, all DK, or the DT and DK metrics together). The cross-validated correlation values at a voxel-based level were low. The best cross-validated correlations were found between DT and DK metrics and the %O.D. MBP. Interestingly, combining DT and DK metrics provided improved prediction of the %O.D. MBP than either DT or DK metrics separately. This high cross-validated correlation value between the DT and DK metrics and the %O.D. MBP contrasted with the low cross-validated correlation values between the DT and DK metrics and the percentage of 4G8 staining, the %O.D. GFAP, and the %O.D. IBA1. This indicates inability to predict amyloid-induced pathology from the DT and DK metrics at a voxel-based level.

## Discussion

In the first part of this study, we investigated if DKI metrics were more predictive of in vivo detection of Aβ-induced pathology than DTI metrics. Of the 23 ROIs investigated, the motor cortex was observed to be the ROI with the highest potential to correctly discriminate APP/PS1 mice from WT mice. From a biological point of view, the latter makes sense because it is a very large ROI with a uniform Aβ pathology. Using LDA, we observed that the accuracy for separate genotypes increased with age (and thus progressing pathology), as well as that a combination of DT and DK metrics offers a better separation of the genotypes than either DT or DK metrics alone. The LASSO analysis indicated that MK and FA were the two most important metrics for this correct classification of the genotype. In addition, MK, AK, RK and FA were best able to predict the underlying Aβ-induced pathological processes as determined by histology. It is noteworthy that we observed high correlations for DKI metrics and FA with the histological parameters when we combined the data from all four time points. However, when we determined the correlations using data from a given time point, the correlations were lower (data not shown). This highlights the need for a longitudinal follow-up study to be able to reliably predict Aβ-induced pathology. Taken together, these results clearly show that, by using an ROI-based approach, acquisition of DKI data has improved potential for the in vivo detection and follow-up of Aβ-induced pathology as compared with when only DTI data is acquired.

In the second part of this study, we investigated if voxel-based statistical parametric mapping would allow us to reliably discriminate APP/PS1 mice from WT mice. We included this type of analysis because it is often used in human studies, is observer-independent, and is less work-intensive than ROI-based analysis. In addition, we correlated the voxel-based DKI metrics to voxel-based histological parameters. Surprisingly, a voxel-based approach was less capable of discriminating APP/PS1 mice from WT mice (compared with an ROI-based analysis) and correlated less with the histologically determined Aβ-induced pathology even when pathology had progressed severely by 8 months of age. A possible explanation for this could be that a voxel at a given location in one animal might contain Aβ plaques, whereas the same voxel in another animal might not. This is a direct result of the biological randomness of where Aβ plaques form within a defined brain region. Therefore, when performing statistical parametric mapping in pre-clinical rodent studies, the latter will cause large variations in the obtained DKI metrics at a voxel level, making it more difficult to statistically discriminate the two genotypes with the statistical models that were used in this study. This drawback is less apparent in human studies, because the voxel size is much larger in humans, and therefore most voxels share the same degree of pathology. For comparison, we used an isotropic voxel size of 78 μm (0.00047 mm^3^), whereas in a study done in humans, Struyfs et al. used an isotropic voxel size of 2.2 mm (10.64 mm^3^) [33]. In contrast, when an ROI-based approach is used, the variation in obtained DKI metrics will be smaller because the degree of pathology is averaged across many voxels. In addition, even small mismatches in the co-registration of the DKI voxels to the histological voxels will have a profound effect on the voxel-based analysis. The ROI-based analysis, however, does not suffer from this drawback. As such, we believe that currently a voxel-based approach is less suitable in pre-clinical rodent studies, whereas it is the method of choice in human studies.

To date, DTI has been used in several human studies to study disease progression in patients with mild cognitive impairment (MCI) and patients with AD. When investigators have looked at the hippocampus in many of these human studies, they have observed reduced FA and increased MD [9, 34–38]. These changes are often attributed to increased extracellular space volume, neurodegeneration and Wallerian degeneration of the white matter. Although we did not see any changes in FA in the hippocampus of APP/PS1 mice, we did observe increased MD (and by extension also axial diffusivity and RD) at 6 and 8 months of age. However, neurodegeneration and Wallerian degeneration are still absent in APP/PS1 mice at 8 months of age [11]. Therefore, we believe the increased MD to be associated primarily with the Aβ induced inflammation (as seen by GFAP and IBA1), which results in oedema and thus an increased extracellular space.

Besides these hippocampal changes, DTI has been able to detect white matter pathology in patients with AD. The latter was initially thought to occur secondary to grey matter damage, but it is now considered to occur separate from grey matter damage, and perhaps even to precede grey matter pathology [39]. We observed reduced FA values in the APP/PS1 mice mainly in the genu and the body of the corpus callosum. This is in concordance with a study by Zerbi et al., who observed this earlier-mentioned increase in DTI metrics in the hippocampus and decreased FA in the genu and body of the corpus callosum [40]. They used 12-month-old APP_swe_/PS1_dE9_ mice, an age at which the plaque load is comparable to what we observe at around 6 months of age in our APP/PS1 mice [41]. A number of human studies indicate that patients with MCI and patients with AD have reduced FA and increased MD in most of the major white matter structures of the brain, and in particular in the corpus callosum, where the changes correlate with the degree of cognitive decline [42–49]. White matter integrity was also found to correlate with CSF Aβ_42_ and phosphorylated tau_181_ [50]. In addition, when patients were followed longitudinally, a further reduction in FA and increase in MD was found in both white matter [51, 52] and grey matter [53] regions. Combined, all of these results indicate the potential of DTI to follow disease progression.

The increased DKI metrics observed in the motor cortex (and by extension in most of the cortical ROIs), as well as the absence of changes in DKI metrics in the hippocampus, are in line with our earlier proof-of-concept study in old APP/PS1 mice [12]. Despite the old age of the APP/PS1 mice in this study (16 months of age), and thus the presence of severe Aβ-induced pathology, the DKI metrics in that study were only modestly increased. In the present study, however, we observed many large changes in DK metrics at very early time points and thus limited Aβ-induced pathology. This increased sensitivity can be ascribed to a further optimisation of our DKI acquisition protocol. Most importantly, Vanhoutte et al. [12] used 30 diffusion gradient directions, whereas in the present study, we used 60 diffusion directions. The latter allows better estimation of the microstructural changes occurring in the brain. To the best of our knowledge, these two studies are the only pre-clinical studies to date in which the usefulness of DKI as a potential biomarker for Aβ-induced pathology has been investigated. Clinically, researchers in only a few recent human studies have examined the usefulness of DKI in this context. For example, Struyfs et al. [33] compared patients with AD with healthy control subjects and reported reduced FA in many regions, among which was the splenium of the corpus callosum. The latter corresponds to our results. However, they also reported reduced MK [33], which contrasts with our finding of an increased MK in many of the cortical ROIs. Reduced MK in the hippocampus of patients with AD was also observed by Wang et al. [54]. We attribute this discrepancy in grey matter findings between the human studies and our pre-clinical work to the fact that our study was focussed on Aβ-induced pathology only, whereas in humans, the whole plethora of pathological events common to AD are present.

One interesting finding was the reduced FA of the septal nuclei in APP/PS1 mice at all ages investigated (2, 4, 6 and 8 months of age). The septal nuclei contain many cholinergic neurons which innervate many of the regions implicated in memory function [55]. Because robust cholinergic dysfunction occurs during AD pathology, many of the currently approved drugs for AD are based on counteracting this loss of cholinergic function in an attempt to restore memory function [56, 57]. As such, the ability of DKI to pick up changes in the septal nuclei even at 2 months of age, when almost no Aβ plaques are present yet, makes DKI very promising for tracking early AD pathology.

## Conclusions

We have demonstrated that for the motor cortex, a combination of DT and DK metrics is more capable of discriminating APP/PS1 mice from WT mice than either DT or DK metrics alone. The power to separate both genotypes also increases as the mice age and thus as Aβ pathology advances, and MK and FA are the two most important metrics for a correct genotype classification. Using a new developed processing platform which enabled co-registration of the in vivo diffusion-weighted MRI with multiple staining of 3D histological stacks, we observed that DKI measures (MK, AK and RK) and FA, but not diffusion measures (MD, axial diffusivity and RD), correlated well with the histologically determined underlying Aβ pathology and the Aβ-induced neuroinflammation. It is noteworthy that we identified the septal nuclei as a region where changes can be visualised very early and thus when Aβ-induced pathology is still largely absent. Lastly, we have also shown that, at least in our hands and in mice, ROI-based analysis is superior to voxel-based analysis. Taken together, our results show that DKI has potential as an in vivo marker for the longitudinal follow-up of Aβ-induced pathology in a transgenic amyloidosis mouse model where atrophy is absent. As such, these results lay the groundwork for translational treatment studies and for the clinical application of DKI to detect early AD pathology in the absence of atrophy.

## Additional files

Additional file 1: The evolution of the weight of the WT and APP/PS1 mice in the longitudinal study at 2, 4, 6, and 8 months of age. (TIF 87 kb)

Additional file 2: An overview of 5 horizontal T2-weighted MRI scans at different levels of depth in the mouse brain on which the 23 different ROIs investigated in this study have been marked. (JPG 1511 kb)

Additional file 3: Graphs showing the Pearson correlations between the MD, AD, and RD with the %O.D. 4G8, GFAP, IBA1, and MBP. The graph includes data from the WT mice (*triangles*) and APP/PS1 mice (*circles*) at 2 months of age (*grey*), 4 months of age (*green*), 6 months of age (*blue*), and 8 months of age (*red*). (JPG 469 kb)

## Abbreviations

Aβ: Amyloid-β
AD: Alzheimer’s disease
ADNI2: Alzheimer’s Disease Neuroimaging Initiative 2 trial
ADNI-GO: Alzheimer’s Disease Neuroimaging Initiative “Grand Opportunities” trial
AK: Axial kurtosis
CSF: Cerebrospinal fluid
DAB: 3,3′-Diaminobenzidine
DK: Diffusion kurtosis
DKI: Diffusion kurtosis imaging
DT: Diffusion tensor
DTI: Diffusion tensor imaging
FA: Fractional anisotropy
FOV: Field of view
FSL: Functional Magnetic Resonance Imaging of the Brain Software Library
4G8: Anti-amyloid-β (clone 4G8) antibody
GFAP: Glial fibrillary acidic protein
IBA1: Ionised calcium-binding adapter molecule 1
IgG: Immunoglobulin G
IHC: Immunohistochemical
LASSO: Least absolute shrinkage and selection operator
LDA: Linear discriminant analysis
MBP: Myelin basic protein
MCE: Misclassification error
MCI: Mild cognitive impairment
MD: Mean diffusivity
MK: Mean kurtosis
MRI: Magnetic resonance imaging
%O.D.: Percent optical density
RARE: Rapid acquisition and relaxation enhancement
RD: Radial diffusivity
RK: Radial kurtosis
ROI: Region of interest
sAβ: Soluble amyloid-β
TE: Echo time
TR: Repetition time
WT: Wild type
