# Supplementary figures and images for: Diffusion kurtosis imaging allows the early detection and longitudinal follow-up of amyloid-β-induced pathology
Source: Alzheimers Res Ther. 2018 Jan 9;10:1. doi: 10.1186/s13195-017-0329-8 (PMC6389136; doi:10.1186/s13195-017-0329-8)

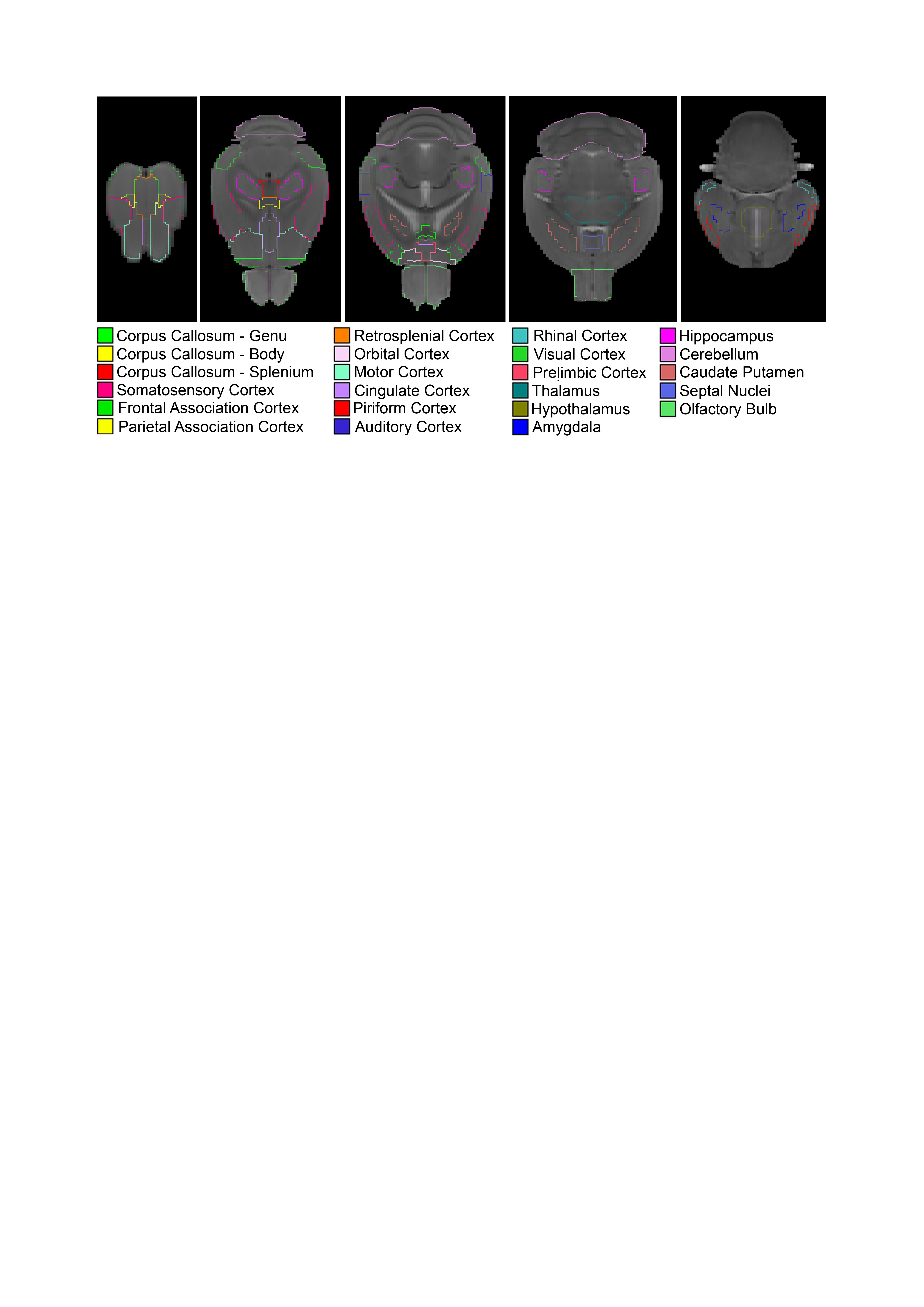

Supplement: Supplementary file 2 — An overview of 5 horizontal T2-weighted MRI scans at different levels of depth in the mouse brain on which the 23 different ROIs investigated in this study have been marked. (JPG 1511 kb) [file 13195_2017_329_MOESM2_ESM.jpg]

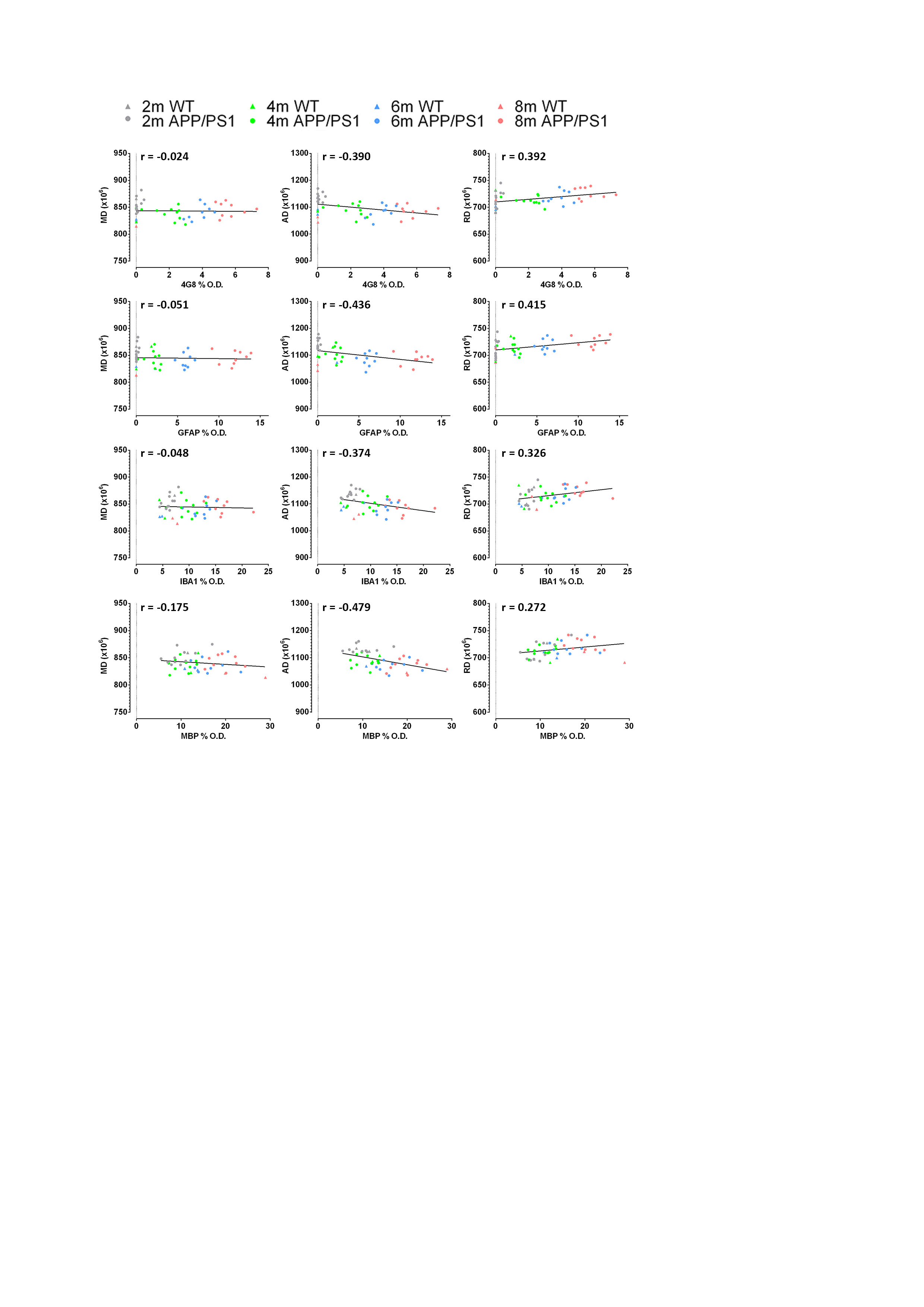

Supplement: Supplementary file 3 — Graphs showing the Pearson correlations between the MD, AD, and RD with the %O.D. 4G8, GFAP, IBA1, and MBP. The graph includes data from the WT mice (triangles) and APP/PS1 mice (circles) at 2 months of age (grey), 4 months of age (green), 6 months of age (blue), and 8 months of age (red). (JPG 469 kb) [file 13195_2017_329_MOESM3_ESM.jpg]
